# First-principles study of the effect of functional groups on polyaniline backbone

**DOI:** 10.1038/srep16907

**Published:** 2015-11-20

**Authors:** X. P. Chen, J. K. Jiang, Q. H. Liang, N. Yang, H. Y. Ye, M. Cai, L. Shen, D. G. Yang, T. L. Ren

**Affiliations:** 1 Institute of Microelectronics, Tsinghua University, 100084 Beijing, China; 2 The Faculty of Mechanical & Electrical Engineering, Guilin University of Electronic Technology, 541004 Guilin, China; 3 Delft Institute of Microsystems and Nanoelectronics, Delft University of Technology, Delft 2628CD, The Netherlands

## Abstract

We present a first-principles density functional theory study focused on how the chemical and electronic properties of polyaniline are adjusted by introducing suitable substituents on a polymer backbone. Analyses of the obtained energy barriers, reaction energies and minimum energy paths indicate that the chemical reactivity of the polyaniline derivatives is significantly enhanced by protonic acid doping of the substituted materials. Further study of the density of states at the Fermi level, band gap, HOMO and LUMO shows that both the unprotonated and protonated states of these polyanilines are altered to different degrees depending on the functional group. We also note that changes in both the chemical and electronic properties are very sensitive to the polarity and size of the functional group. It is worth noting that these changes do not substantially alter the inherent chemical and electronic properties of polyaniline. Our results demonstrate that introducing different functional groups on a polymer backbone is an effective approach to obtain tailored conductive polymers with desirable properties while retaining their intrinsic properties, such as conductivity.

Conductive polymers exhibit the electrical, electronic, magnetic, and optical properties of a metal or semiconductor while retaining the advantages of a conventional polymer, such as favourable mechanical properties and solution processability^1^. Conductive polymers have therefore attracted considerable attention since the initial discovery of polyacetylene (CH)_*x*_ in 1977^2^,^3^. Polyaniline holds a special position amongst conductive polymers because of its ease of synthesis, low cost, environmental stability, and unique doping/dedoping mechanism^3^. According to the Mott’s variable-range hopping mechanism^4^, the 3D *dc* conductivity of protonated polyaniline is given by:


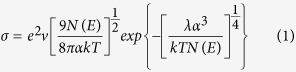


where *σ* is the *dc* conductivity as a function of the thermodynamic temperature *T*; *ν*, *α* and *N*(*E*) represent the hopping frequency, inverse rate of the fall of the wave function and the density of states (DOS) at the Fermi level^5^, respectively; and *e*, *k* and *λ* (≈18.1) denote the electronic charge, Boltzmann’s constant and a dimensionless constant, respectively. While polyaniline has been widely used in nanoelectronics^6^, nanosensors^7^, nanomaterials (e.g., nanowires^8^ and nanofibres^9^), its applications are limited by a number of issues, such as poor solubility in most available organic solvents^10^ and weak chemical reactivity of protonic acid doping, which can only occur in a relatively strongly acidic environment (pH < 4.0)^11^. Recent studies^8^,^11^,^12^,^13^ have shown that the introduction of suitable substituents, such as sulfonic acid (-SO_3_Na or -SO_3_K)^8^,^11^, boronic acid (-BO_2_H_2_)^12^, and carboxylic (-COONa)^13^ groups, at the phenyl rings or nitrogen sites of polyaniline is the simplest and most cost-effective approach for addressing these issues. However, the impact of derivatization at the phenyl ring and nitrogen sites at the atomic level on the chemical and electronic properties necessary for the design of polyaniline for practical applications remains unclear.

A promising solution to address these challenging issues is the application of molecular-based computational approaches. Conductive polymers for various applications can be evaluated and selected using purely computational approaches, which have generally focused on the development of a fundamental electronic- and atomic-level description to provide insight into the structure and properties of materials^14^,^15^,^16^,^17^,^18^,^19^. For instance, Varela-Álvarez *et al.*^16^,^17^ evaluated the effect the chain length and number of monomers on the electronic properties of polyaniline. Using density-functional theory (DFT) simulations, Wang *et al.*^18^ systematically studied the stability and electronic structure of HCl-protonated polyaniline and polyaniline–graphene composites. Romanova *et al.*^19^ computationally analysed polyaniline–water interactions by employing a polarizable continuum model. However, to date, there have been no theoretical studies on the effects of the functional groups on a polyaniline backbone.

In this work, we perform a detailed first-principles study to explore how derivatization at the polymer chain using suitable aromatic substituents, such as –OH and –SO_3_Na groups, affects both the chemical and electronic properties of polyaniline. For this purpose, we first examine the energy barrier (*E*_*bar*_), reaction energy (*E*_*r*_) and minimum energy path for carbonic acid (H_2_CO_3_) doping of these polyanilines to provide insight into how their chemical properties depend on their molecular structures. In this study, *E*_*bar*_ refers to the energy difference between the intermediate (the state corresponding to the highest energy along the reaction coordinate) and the reactant in H_2_CO_3_ protonation reactions. Additionally, *E*_*r*_ denotes the difference between the product energy and the reactant energy. To determine whether the intrinsic conductivity of the resulting substituted polyanilines has been changed, we calculate the density of states at the Fermi level *N*(*E*) and the band gap for each of the studied polymers. To obtain deeper insight into the dependence of the electronic properties on the polymer structures, we compare the spatial distribution of the highest occupied molecular orbital (HOMO) and the lowest unoccupied molecular orbital (LUMO) for all unprotonated and H_2_CO_3_-protonated polyanilines, respectively. The present investigation is the first theoretical prediction of the effect of the functional groups on a polyaniline backbone obtained using first-principles calculations.

## Molecular Models and Computational Details

In this work, a half-oxidized polymer^1^, emeraldine base of polyaniline (EB-PANI), was used as the parent polyaniline. Direct derivatization at the polyaniline chain was performed by introducing one functional group (–OH or -SO_3_Na) for every monomer, and the resulting substituted polymers were denoted as HO-PANI and Na-SPANI, respectively. Protonic acid doping of these polyanilines was achieved by equilibrating them with the weak acid H_2_CO_3_. Two polymer models were used to predict the polymer properties: finite molecular models and periodic molecular models.

### Predictions using finite molecular models

**Figure 1:**
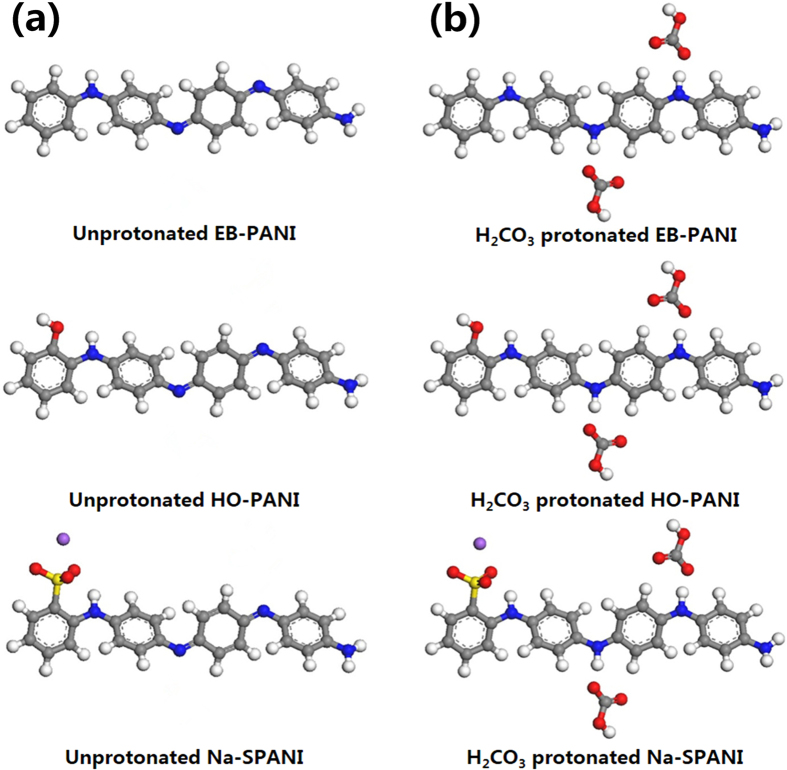
The finite molecular models with one polymer monomer for EB-PANI, HO-PANI and Na-SPANI: (a) Unprotonated and (b) H_2_CO_3_-protonated states. The hydrogen, carbon, nitrogen, oxygen, sulfur and sodium atoms are shown in white, grey, blue, red, yellow and violet, respectively.

The finite molecular models for these polyanilines in their unprotonated and H_2_CO_3_-protonated states are schematically represented in Fig. 1(a,b), respectively. Each finite molecular model consists of one polymer monomer. The calculations of the energy barrier *E*_*bar*_, reaction energy *E*_*r*_, and the HOMO and LUMO energies for the finite molecular models were performed using the DMol^3^ package of Materials Studio^®^ 7.0 (Accelrys, San Diego, CA, USA) and dispersion-corrected DFT (DFT-D). To fully optimize the finite molecular models, the standard Perdew-Burke-Ernzerhof (PBE) generalized gradient approximation (GGA)^20^ with Grimme’s long-range dispersion correction^21^ was used for the exchange-correlation functional. The double numeric plus polarization (DNP) basis set was employed for all calculations. The convergence tolerance of the energy was set to 10^−5^ hartree (1 hartree = 27.21 eV), and the maximal allowed displacement and force were 0.005 Å and 0.002 hartree/Å, respectively. The linear synchronous transit/quadratic synchronous transit (LST/QST) and nudged elastic band (NEB) tools were used to investigate the minimum energy pathway for the H_2_CO_3_ doping of EB-PANI and its derivatives.

### Prediction using periodic molecular models

**Figure 2:**
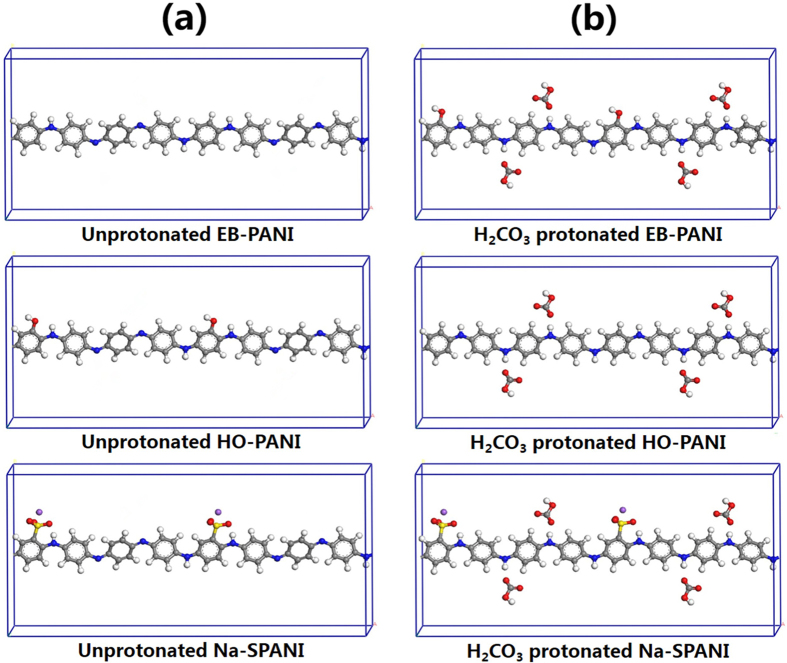
The periodic molecular models with two polymer monomers for EB-PANI, HO-PANI and Na-SPANI: (a) Unprotonated and (b) H_2_CO_3_-protonated states.

The periodic molecular models for polyanilines in unprotonated and H_2_CO_3_-protonated states are depicted in Fig. 2(a,b), respectively. All periodic molecular models were generated using one polymer chain with two monomers surrounded by vacuum. To suppress the surface effects and use a reasonable number of particles in the model, 3D periodic boundary conditions (PBC) were employed. For our periodic systems, the calculations of the density of states at the Fermi level *N*(*E*) and the band gap *E*_*g*_ were also performed using the DMol^3^ package and DFT-D based on GGA-PBE. The *k*-point grid was set to be 8 × 1 × 1. A tighter smearing criterion of 0.01 hartree was employed to improve the electronic convergence. To consider the temperature effect on the electronic properties as addressed in our previous study^11^,^22^, the initial optimization of the periodic molecular model by DMol^3^ was followed by running a canonical ensemble-molecular dynamics (NVT-MD) for 50 ps (time step = 0.001 ps) at room temperature (298 K) using the Forcite^23^ package of Materials Studio, and the commercial force field “COMPASS II” was used to evaluate the atomic forces. The temperature was controlled by the “Berendsen” method using a half-life for decay to the target temperature in 0.1 ps. The summation methods “atom-based” with a cutoff value of 12.5 Å and “Ewald” were employed for the non-bonded van der Waals (vdW) and electrostatic (or Coulomb) interactions, respectively.

## Results and Discussion

### Chemical reactivity

**Figure 3:**
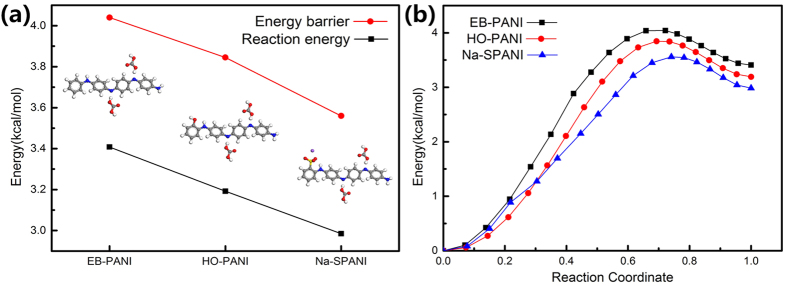
Chemical reactivity of H_2_CO_3_ doping of EB-PANI, HO-PANI and Na-SPANI according to finite molecular models: (a) Reaction energy *Er* and energy barrier *E*_*bar*_ and (b) the minimum energy paths with initial energy at zero.

Protonic acid doping is a unique chemical property of polyaniline. Therefore, we determined how the chemical properties depend on the different functional group by quantifying the chemical reactivity (*E*_*r*_ and *E*_*bar*_) using the weak acid H_2_CO_3_ to protonate each polymer. As plotted in Fig. 3(a), the energy barrier *E*_*bar*_ values for H_2_CO_3_ doping of EB-PANI, HO-PANI and Na-SPANI are 4.04, 3.85 and 3.56 kcal/mol, respectively. The Arrhenius equation describes the temperature dependence of reaction rates. The Arrhenius relation for protonation reaction^18^ is given by:


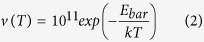


where *ν*(*T*) is the rate constant at the given temperature and *E*_*bar*_, *k* and *T* are the energy barrier, Boltzmann’s constant and thermodynamic temperature, respectively. Here, the *E*_*bar*_ (when 1/*ν*(*T*) ∼ 1.0)^18^ value for the H_2_CO_3_ doping reaction is estimated to be 14.99 kcal/mol (this magnitude is considered surmountable for H_2_CO_3_ protonation reactions occurring at room temperatures). Thus, the energy barriers for H_2_CO_3_ doping of EB-PANI, HO-PANI and Na-SPANI are relatively low. We therefore deduce that these polyanilines could be protonated by H_2_CO_3_ at room temperature. It is evident from Fig. 3(a) that there is a surprising difference between *E*_*bar*_ values and that the *E*_*bar*_ decreases in the order of *E*_*bar* (EB-PANI)_ > *E*_*bar* (HO-PANI)_ > *E*_*bar* (Na-SPANI)_. The reaction energy *E*_*r*_ values for H_2_CO_3_ doping of EB-PANI, HO-PANI and Na-SPANI are also plotted in Fig. 3(a) and are 3.41, 3.19 and 2.99 kcal/mol, respectively. The *E*_*r*_ values of the three polymers follow the same order as the decrease of the *E*_*r*_ values. Remarkably, we find that both *E*_*bar*_ and *E*_*r*_ are significantly reduced upon the introduction of -OH and -SO_3_Na functional groups on the phenyl rings. We also observed that both *E*_*bar*_ and *E*_*r*_ decreased with as the size and polarity of the functional groups increase. To further analyse the changes in the chemical properties, we extended the study by investigating the reaction paths presented in Fig. 3(b). Clearly, the introduction of functional groups decreases *E*_*bar*_, thereby increasing the likelihood of protonic acid doping. The above results of *E*_*bar*_, *E*_*r*_ and the minimum reaction paths imply that protonic acid doping of HO-PANI and Na-SPANI may occur at higher pH than EB-PANI. A similar result was observed in the experimental study by Cees *et al.*^8^, which was focused on developing polyaniline-based carbon dioxide (CO_2_) sensors. They found that the protonation of EB-PANI is complete only at pH values lower than 4, whereas Na-SPANI can also be protonated in the pH 4–7 range. Therefore, we propose that the chemical properties, especially the chemical reactivity, of polyaniline are very sensitive to derivatization at the polymer chain and that the order of the chemical reactivity for protonic acid doping increases from EB-PANI to HO-PANI to Na-SPANI.

### Electronic properties

**Figure 4:**
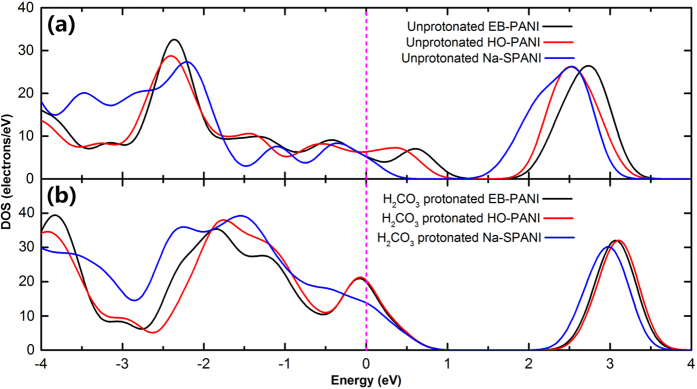
Density of states of EB-PANI, HO-PANI and Na-SPANI according to periodic molecular models: (a) unprotonated and (b) H_2_CO_3_-protonated states. The vertical dashed lines represent the Fermi level, and the electronic convergence is accelerated with smearing the Fermi surface by 0.01 hartree.

Conductive polymers, specifically intrinsically conducting polymers, are polymeric organic materials that are intrinsically conducting^1^. According to Mott’s theory, which is summarized by Equation (1), the *dc* conductivity^24^ of polyaniline at a given temperature is determined by the DOS at the Fermi energy *N*(*E*)^7^. Therefore, to examine whether the intrinsic conductivity of polyaniline will be substantially changed by the presence of functional groups, we calculated the DOS and compared the *N*(*E*) for all the polymers. The DOS of the relaxed polymers in unprotonated and H_2_CO_3_-protonated states are given in Fig. 4(a,b), respectively, with the vertical dashed lines indicating the *N*(*E*) position. Comparing *N*(*E*) values of the different polyanilines in the unprotonated state, as shown in Fig. 4(a), clearly shows that there is little difference between their *N*(*E*) values, *i.e.*, the presence of functional groups does not substantially change the electronic properties of the parent polyaniline. However, comparing the *N*(*E*) values with those of their carbonate salts, as shown in Fig. 4(b), demonstrates that the *N*(*E*) of polyaniline in the protonated state is more sensitive to -SO_3_Na than to -OH. What is the origin of this difference? The possible underlying factors are the polarity and size of the functional group. The *N*(*E*) value represents the electronic properties of microscopic polyaniline in Equation (1); hence, we consider the conductivity of Na-SPANI in protonated state to be worse than that of the carbonate salts of EB-PANI and HO-PANI. This deduction can be verified using the experimental result of MacDiarmid *et al.*^25^, who showed that the conductivity of the hydrochloric acid (HCl)-protonated Na-PANI is decreased by approximately one order of magnitude relative to that of HCl-protonated EB-PANI. To gain deeper insight into their electronic properties, the band gaps of these polymers in unprotonated and H_2_CO_3_-protonated states are also analysed, as listed in Table 1. However, it should be noted that the difference in the band gap remains fairly small, and the band gap values maintain the same magnitude, especially for the carbonate salts.

### HOMO and LUMO

**Figure 5:**
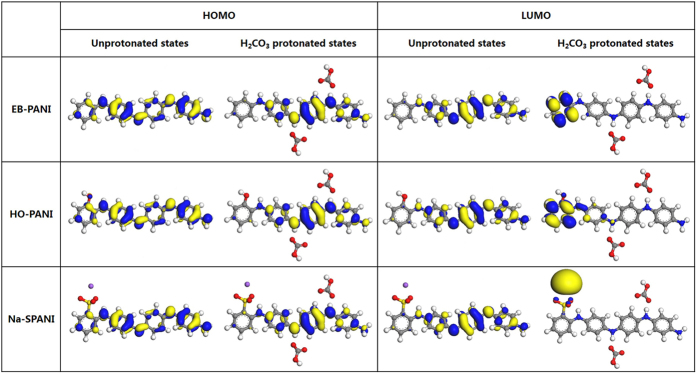
Comparison between the HOMO and LUMO of the unprotonated and H_2_CO_3_-protonated states of EB-PANI, HO-PANI and Na-SPANI according to finite molecular models.

The HOMO/valence band and LUMO/conduction band of *p*-conjugated molecules/polymers are generally derived from occupied *p*-bonding orbitals and unoccupied p*-antibonding orbitals, respectively^26^. The difference between the valence band maximum (VBM) and the conduction band minimum (CBM) is called the band gap^26^,^27^,^28^. To obtain further insight into the chemical and electronic properties of these polyanilines, we investigate the spatial distribution of the HOMO and LUMO for all polyanilines and their carbonate salts, and the calculated results are shown in Fig. 5. For all unprotonated polyanilines, the HOMO delocalizes across the whole polymer backbone and mainly surrounds the benzenoid rings, while the LUMO is mainly distributed at the quinoid ring. It is evident that the distributions of both the HOMO/valence band and LUMO/conduction band in the polyanilines show very small changes. In other words, introducing the functional groups on the phenyl rings does not substantially change the inherent chemical properties (e.g., protonic acid doping mechanism) and the electronic properties (e.g., nonconductor in unprotonated state) of the parent polyaniline. For the protonated polyanilines, the HOMO mainly localizes around the reduced quinoid ring between the two –(NH)^+^HCO_3_^−^ dipoles and shows no response to the various functional groups; in contrast, obvious differences are observed for the LUMO. In H_2_CO_3_-protonated EB-PANI, the LUMO is almost completely localized around the first benzenoid ring, away from the –(NH)^+^HCO_3_^−^. However, in H_2_CO_3_-protonated HO-PANI, the LUMO mainly localizes around the first benzenoid ring and is also slightly distributed in the amine links. We suggest that the –OH functional group draws the electron cloud of the first benzene ring toward itself; then, the electron cloud of the amine links creates an offset for the benzene ring, eventually resulting in a slight LUMO distribution in the amine links. Thus, higher LUMO character in the H_2_CO_3_-protonated HO-PANI backbone can upshift the LUMO and make the band gap larger than that in the H_2_CO_3_-protonated EB-PANI. Compared with the carbonate salts of EB-PANI and HO-PANI, the LUMO of the H_2_CO_3_-protonated Na-SPANI is found almost entirely on the Na^+^ cation, with a slight presence on the -SO_3_^–^. We suggest that the Na atom contributes its electrons to the combination of the first benzenoid ring and –SO_3_^–^, resulting in a supersaturation state. Therefore, the LUMO is substantially downshifted, decreasing the band gap further than in EB-PANI and HO-PANI carbonates. Based on the above results, we conclude that the functional group has a rather weak influence on the distributions of the HOMO and LUMO in the unprotonated polyanilines, whereas it will affect the distribution of the LUMO in the protonated polyanilines to some extent.

## Conclusions

In conclusion, we performed an *ab initio* study to elucidate the effect of direct functionalization of a polyaniline backbone on polyaniline properties by employing DFT. We demonstrated that the introduction of –OH and –SO_3_Na groups at the phenyl rings leads to a remarkable improvement in the chemical reactivity for the protonation of polyaniline by equilibration with H_2_CO_3_. We also observed that the hybridization between the functional group and the phenyl ring induces an impurity state near the Fermi level and band gap changes in the carbonate salts of polyanilines. Another important finding is that the distributions of the HOMO and LUMO in unprotonated polyanilines are relatively insensitive to the functional group, while the distribution of the LUMO in the carbonates exhibits obvious changes depending on the functional group. We propose that the differences in the distribution of the LUMO alter the band gap in the protonated states. Comparative studies of –OH and –SO_3_Na indicate that both chemical and electronic properties are very sensitive to the polarity and size of the functional groups. Most importantly, the presence of a functional group on the phenyl ring does not substantially change the inherent chemical and electronic properties of the parent polyaniline. Our findings provide a basis for understanding the role played by derivatization at the polyaniline backbone in affecting the chemical properties, doping and conducting mechanism of polyaniline. The present investigation also provides an effective pathway to design conductive polymers with desired properties without substantially changing the intrinsic properties, such as conductivity, to satisfy the requirements of various applications.

## Additional Information

**How to cite this article**: Chen, X. P. *et al.* First-principles study of the effect of functional groups on polyaniline backbone. *Sci. Rep.* **5**, 16907; doi: 10.1038/srep16907 (2015).

## Figures and Tables

**Table 1:** The band gap values of EB-PANI, HO-PANI and Na-SPANI in unprotonated and H_2_CO_3_-protonated sates.

Type	Band gap (eV)	
	Unprotonated states	H_2_CO_3_-protonated states
EB-PANI	0.51	0.163
HO-PANI	0.306	0.193
Na-SPANI	0.538	0.122
